# Fear of Fragility: A Case of Osteoporosis-Triggered Takotsubo Cardiomyopathy

**DOI:** 10.7759/cureus.75289

**Published:** 2024-12-07

**Authors:** Admire Hlupeni, Wagmah J Khan, Adebisi Adejola, Shane J LaRue

**Affiliations:** 1 Internal Medicine, St. Luke's Hospital, Chesterfield, USA; 2 Immunology, Midlands State University, Gweru, ZWE; 3 Cardiology, St. Luke's Hospital, Chesterfield, USA

**Keywords:** emotional well-being, osteoporosis, primary care clinic, spikes, takotsubo cardiomyopathy

## Abstract

We present a case of a 73-year-old woman with a medical history significant for hyperlipidemia, on pravastatin, who developed Takotsubo cardiomyopathy following a diagnosis of osteoporosis.

She presented to the Emergency Department with acute transient left arm pain that resolved spontaneously. Investigations revealed elevated troponin levels, non-specific electrocardiographic changes, no significant coronary artery disease on angiography, and left ventricular systolic dysfunction, findings consistent with Takotsubo cardiomyopathy. Further evaluation revealed that the patient’s intense anxiety about her recent osteoporosis diagnosis served as the primary emotional stressor, ultimately triggering Takotsubo cardiomyopathy.

Osteoporosis, though not immediately life-threatening, can evoke significant psychological distress due to fears about future fractures, physical limitations, and loss of independence. This highlights the broader impact of chronic disease diagnoses on emotional well-being, particularly in older adults. Management in this case included losartan, carvedilol, and supportive care. Beyond pharmacologic treatment, addressing the patient’s emotional health was a priority. Specific psychosocial interventions included in-depth discussion with the patient to alleviate misconceptions about osteoporosis, referrals to counseling services to manage anxiety, and strategies to build coping mechanisms such as mindfulness and relaxation techniques. Providing education on the manageable nature of osteoporosis and available treatments helped reduce the patient’s sense of helplessness. These interventions aimed to not only support the patient’s immediate recovery but also to minimize the risk of recurrence of stress-induced adverse events such as Takotsubo cardiomyopathy.

The case underscores the importance of integrating emotional well-being into routine clinical practice by offering tailored psychosocial support, including clear communication, access to mental health resources, and fostering a collaborative care environment. Holistic care that addresses both physical and psychological health can improve patient outcomes, reduce hospitalizations, and empower patients to manage their conditions more effectively.

## Introduction

Takotsubo cardiomyopathy, also called stress-induced cardiomyopathy or broken heart syndrome, is a syndrome characterized by transient regional systolic dysfunction, principally of the left ventricle [1]. It is an increasingly recognized condition with a prevalence of approximately 0.02% of all hospitalizations in the United States and its incidence has shown a significant increase over recent years, likely due to heightened awareness and improved diagnostic capabilities [2,3].

It mimics myocardial infarction but occurs without angiographic evidence of obstructive coronary artery disease [4,5]. Patients typically present with symptoms similar to acute coronary syndrome, including chest pain and shortness of breath, along with electrocardiographic changes and elevated cardiac injury biomarkers [6]. Although Takotsubo cardiomyopathy has been extensively studied since its discovery, its pathophysiology remains unclear. It is thought to involve a surge in catecholamines, leading to myocardial stunning, microvascular dysfunction, and direct myocardial injury [4-6]. Literature also suggests that different hormones, such as steroids, also play a role in the pathogenesis of Takotsubo cardiomyopathy [7-9]. Identified risk factors include postmenopausal status, emotional or physical stress (such as grief, fear, acute illness, or surgery), and psychological conditions like anxiety and depression [4-6,10]. Management typically involves cardiac catheterization to exclude obstructive coronary disease, along with the use of beta-blockers and angiotensin-converting enzyme inhibitors. In cases where a left ventricular thrombus is present, anticoagulation therapy may also be necessary [11].

We present a case of Takotsubo cardiomyopathy in a 73-year-old postmenopausal woman, triggered by an unexpected stressor of receiving a diagnosis of osteoporosis at a primary care clinic. While Takotsubo cardiomyopathy is often linked to severe emotional or physical stressors, such as the loss of a loved one, this case highlights a less commonly recognized trigger. Osteoporosis, typically considered a routine medical diagnosis, is particularly relevant in postmenopausal women, as it can provoke significant anxiety due to its implications for fracture risk, reduced mobility, and potential loss of independence. The patient provided written informed consent for this case to be documented and submitted for publication.

## Case presentation

The case involved a 73-year-old woman with hyperlipidemia, managed with pravastatin 40 mg daily, and a diagnosis of age-related osteoporosis, identified three months ago without associated fractures. She had been prescribed alendronate but had not initiated the medication. She presented to the Emergency Department (ED) with acute left arm pain that had started about two hours before, which woke her from sleep at 5 AM on the morning of presentation. The pain, described as severe and accompanied by numbness, originated in the left arm and spread to the left scapula, across the upper chest, and to the right upper arm. Symptoms resolved spontaneously about an hour after onset, prior to receiving any treatment. She denied prior similar episodes, palpitations, shortness of breath, nausea, vomiting, abdominal pain, diarrhea, dysuria, cough, fever, or chills.

Past medical history

Her past medical history was notable for the absence of cardiovascular disease, including hypertension and coronary artery disease. Her surgical history included a hysterectomy and laparoscopic adhesiolysis over 10 years ago. She was not on any other regular medication besides pravastatin.

Family history 

Family history revealed diabetes in her father, hypertension in her mother, and stroke in her sister.

Social history

She had never smoked and drank alcohol occasionally. She was in a stable marriage and living with her husband.

ED findings

In the ED, her vital signs included a temperature of 36.6°C, blood pressure of 172/72 mmHg, and a heart rate in the 80s-90s. BMI was 23.56, and oxygen saturation was 98% on room air. Physical examination was unremarkable, with a supple neck, no jugular venous distension, normal heart sounds, clear lungs, a soft, non-tender abdomen, and no extremity edema.

**Figure 1:**
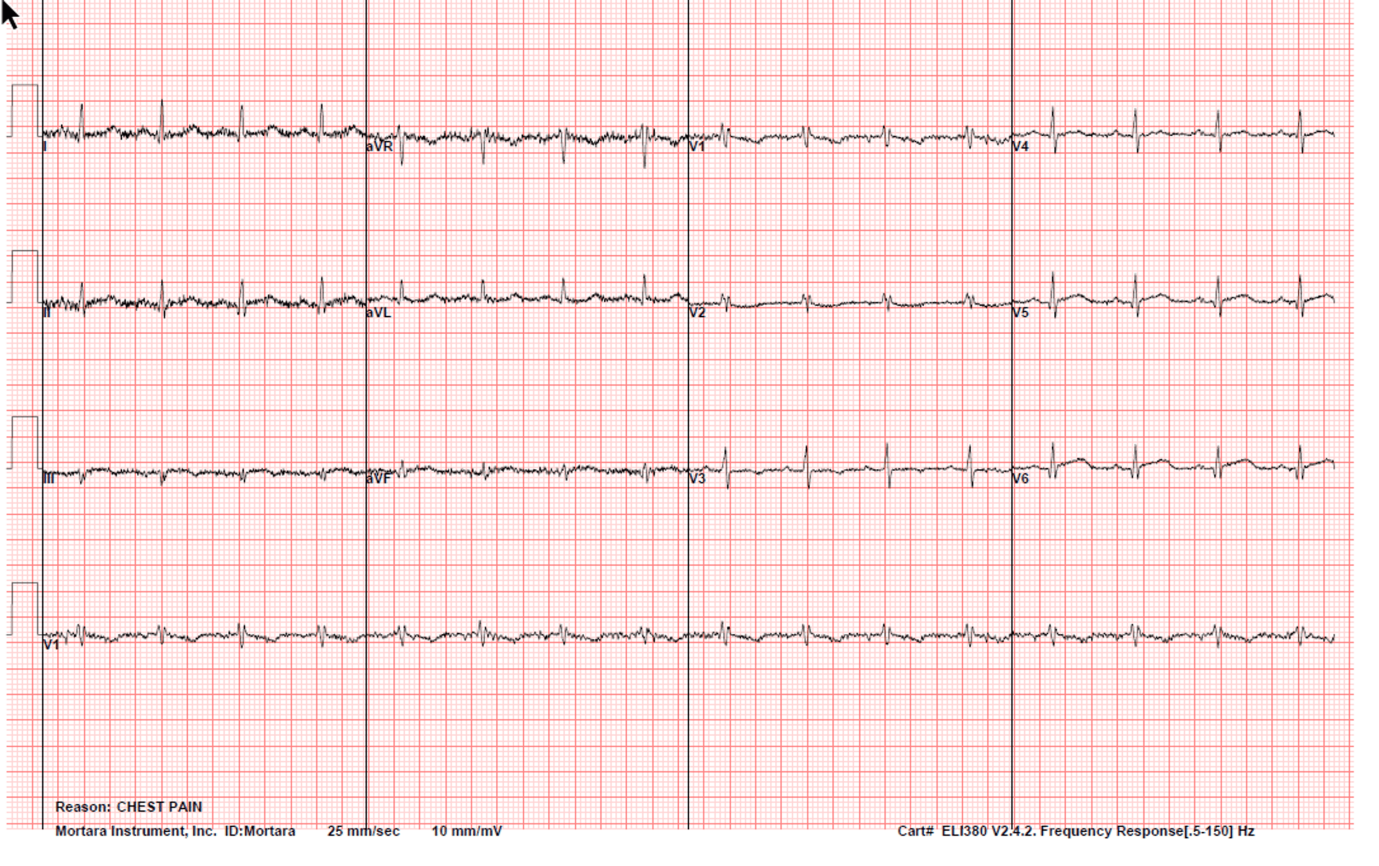
An electrocardiogram (ECG) conducted on the day of presentation to the Emergency Department, showing regular sinus rhythm at a ventricular rate of 95 beats per minute, low voltage in leads III, aVF, V1, and V2, non-specific ST and T abnormalities in leads III, aVF, and V1-V3, and possible right ventricular conduction delay.

**Figure 2:**
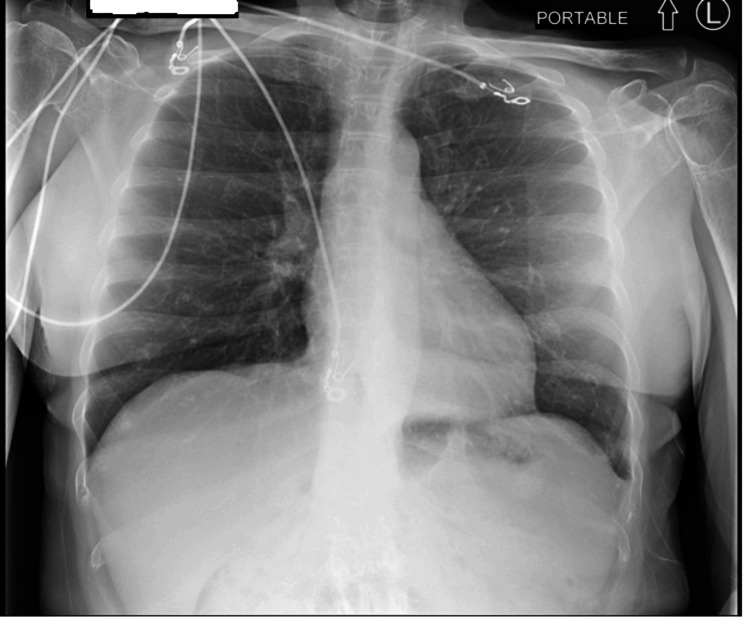
Antero-posterior chest X-ray image obtained on the day of presentation to the Emergency Department, showing no acute cardiopulmonary abnormalities.

**Figure 3:**
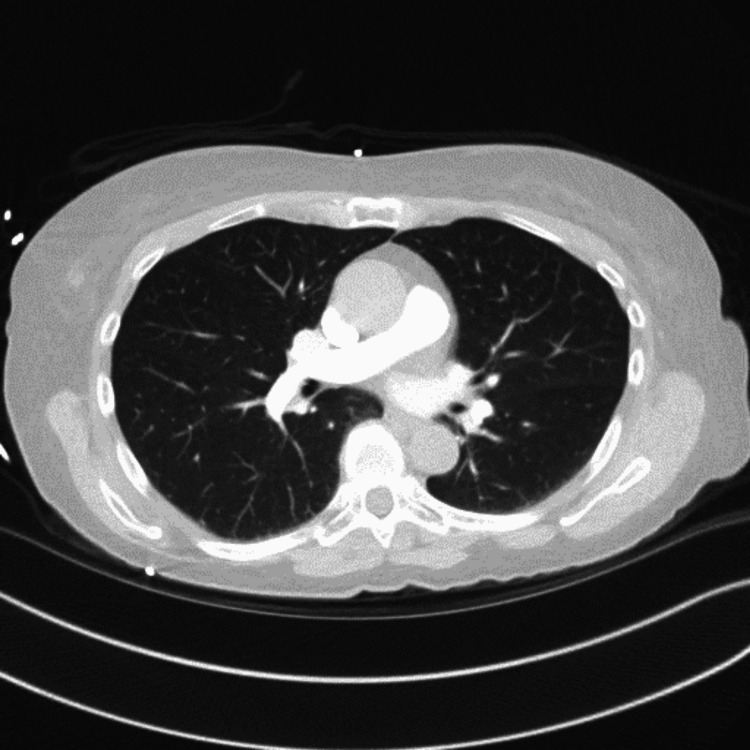
An image from an axial computed tomography (CT) angiography pulmonary embolism protocol conducted on the day of presentation to the Emergency Department, showing no evidence of pulmonary embolism or acute cardiopulmonary abnormalities.

An electrocardiogram (ECG) (Figure 1) revealed regular sinus rhythm, low voltage in leads III, aVF, V1, and V2, possible right ventricular conduction delay, and non-specific ST and T wave abnormalities in leads III, aVF, and V1- V3. Laboratory results (Table 1) revealed elevated troponin I levels (0.85), while proBNP (49 pg/mL), sodium, potassium, blood urea nitrogen, creatinine, alanine transaminase, aspartate aminotransferase, white blood cell count, hemoglobin, and platelets were within normal ranges. Repeat troponin I measurements at two and six hours showed a rising trend, increasing to 3.65 and 10.40, respectively. These samples were collected prior to the patient's coronary catheterization, and no further measurements were taken afterward. Chest X-ray (Figure 2) showed no acute cardiopulmonary abnormalities, and CT angiography showed no evidence of pulmonary embolism or acute cardiopulmonary abnormalities (Figure 3).

**Table 1:** Laboratory testing conducted for the patient on presentation to the Emergency Department and during her hospital stay. Day 1 is the day the patient presented to the Emergency Department. ALT: Alanine transaminase; AST: Aspartate aminotransferase; BUN: Blood urea nitrogen; eCrCl: Estimated creatinine clearance; POC: Point of care; MCV: Mean corpuscular volume; WBC: White blood cells.

Time of sample collection	Reference ranges and/or units	Day 2 at 0406 hours	Day 1 at 1305 hours	Day 1 at 0905 hours	Day 1 at 0654 hours
Hematology					
WBC	4.3-10.0 K/ul	5.4	7.8		4.8
Hemoglobin	11.8-14.8 g/dl	13.6	13.5		15.0
MCV	82- 99.0 fl	94.0	97.3		93.5
Platelet count	140-350 K/ul	185	206		217
Chemistry					
Sodium	137-145 mmol/l	140			141
Potassium	3.4-5.1 mmol/l	3.8			3.9
Chloride	98-107 mmol/l	108			105
Carbon dioxide	22-30 mmol/l	25			24
BUN	7-17 mg/gl	12			22
Creatinine	0.5-1 mg/dl	0.72			0.78
Glucose	74-106 mg/dl	98			110
Calcium	8.4-10.2 mg/dl	8.6			9.2
Albumin	3.5-5.0 g/dl				4.9
Alkaline phosphatase	38-126 U/l				89
Total bilirubin	0.2-1.3 mg/dl				0.8
AST	14-42 U/l				49
ALT	≤ 35 U/l				38
eCrCl (C-Gault)	ml/min/kg	1.1			1.0
Troponin I	Critical high >0.12		10.40	3.65	0.85
Troponin (POC-iSTAT)	Critical high >0.08				0.58
Magnesium	1.6-2.6 mg/dl	2.3			
NT-proBNP	pg/ml				49

Differential diagnoses

Based on the chest symptoms, ECG findings, and elevated troponin I levels, the initial diagnosis was most likely non-ST-elevation myocardial infarction (non-STEMI), in the context of newly diagnosed hypertension and pre-existing hyperlipidemia. The normal pro-BNP level (49 pg/mL) effectively ruled out acute decompensated heart failure. At this point, Takotsubo cardiomyopathy was not considered, as there were no evident life-threatening events or commonly associated stressors typically linked to the condition.

Management

**Figure 4:**
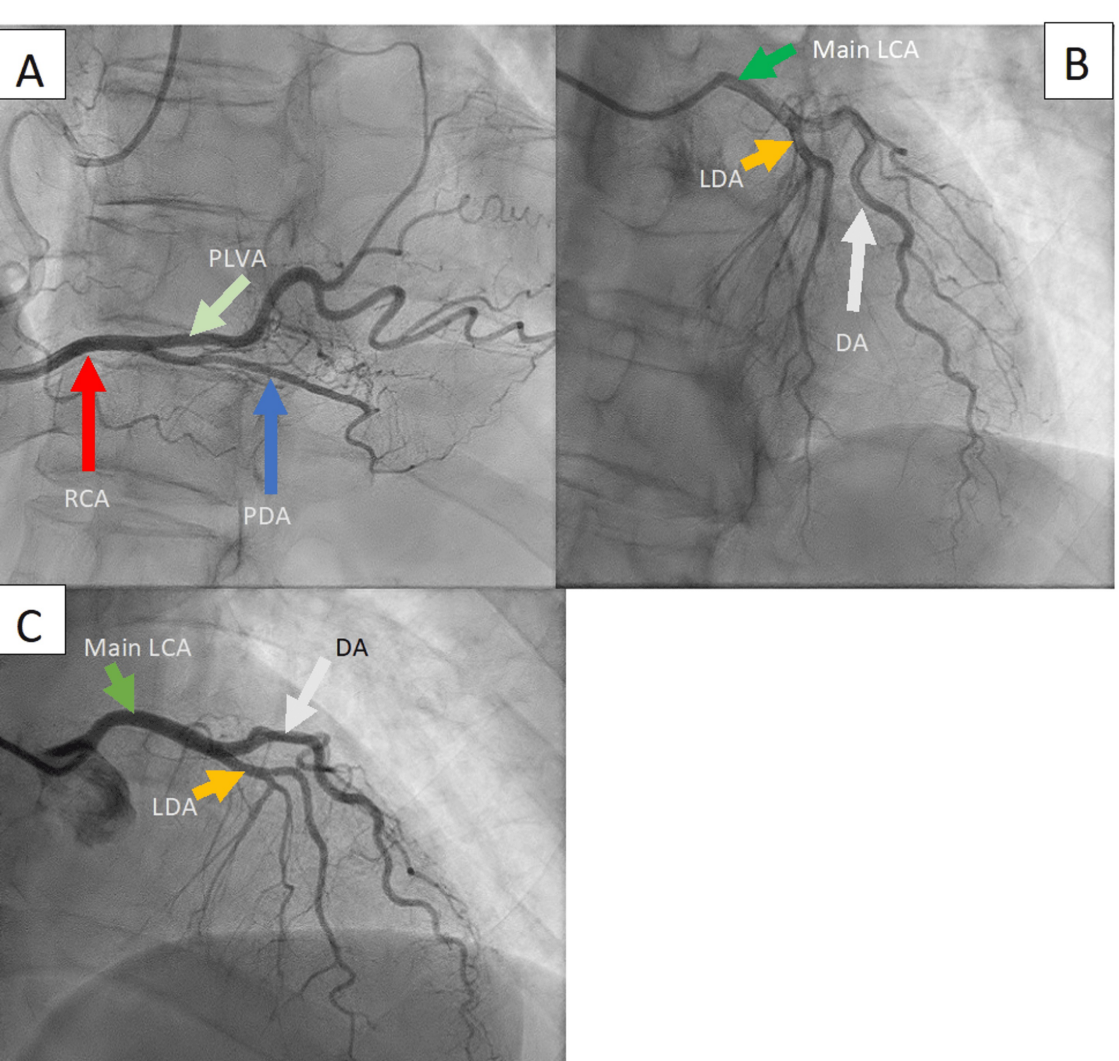
Images from the cardiac catheterization showing grossly normal and patent right (panel A) and left (panels B and C) coronary arteries. Note the complete absence of the circumflex artery in accordance with a large anterolateral diagonal branch and a super dominant right coronary artery. RCA: Right coronary artery; PLVA: Posterior left ventricular artery; PDA: Posterior descending artery; LCA: Left coronary artery; DA: Diagonal artery; LAD: Left anterior descending

She received stat doses of aspirin 324 mg, nitrates 0.4 mg, and heparin 4000 units and was admitted to the telemetry ward. The patient then underwent a coronary catheterization (Figure 4) which was performed about eight hours after presentation. It showed a right-dominant coronary artery system. The left main and right coronary arteries were free of significant disease. The left anterior descending artery was diminutive with mild distal luminal irregularities, while the circumflex artery was absent, replaced by a large anterolateral diagonal branch and a super-dominant right coronary artery. This effectively ruled out a non-STEMI diagnosis. Assessment of the left ventricular systolic function showed dysfunction, with distal anterolateral, apical, and infero-apical hypokinesis and an ejection fraction of 35-40%, findings consistent with Takotsubo cardiomyopathy. No evidence of a clot was found in the LV.

Following the diagnosis of Takotsubo cardiomyopathy, we engaged in a detailed discussion with the patient to explore potential triggers. She revealed significant emotional distress and an overwhelming fear of fractures following her osteoporosis diagnosis. This anxiety had led to severe inactivity and reluctance to start alendronate due to concerns about potential side effects. Her fear of fracturing her bones was so profound that she largely confined herself to bed or the couch. To address these concerns, we had an in-depth conversation to dispel misconceptions about osteoporosis and emphasized its manageable nature. We involved hospital counselors to help manage her anxiety and fear, and recommended strategies to build coping mechanisms, such as mindfulness and relaxation techniques. Additionally, we provided education on the available treatment options, which helped alleviate her sense of helplessness and vulnerability. She also agreed to follow up with her primary care provider (PCP) after hospital discharge to address any other questions or concerns regarding the osteoporosis or its treatment. She was also started on losartan 12.5 mg once daily and carvedilol 3.125 mg twice daily, in line with recommendations for managing Takotsubo cardiomyopathy [11]. She was not continued on aspirin during the hospital stay.

The patient remained asymptomatic during her hospital stay and showed emotional improvement. She was discharged on day three with instructions to follow up with her PCP and cardiologist as an outpatient. We do not have data on follow-up laboratory tests or imaging, including repeat echocardiography, after the patient’s discharge from the hospital.

## Discussion

We presented a case of Takotsubo cardiomyopathy in a 73-year-old postmenopausal woman, triggered by an unexpected stressor: receiving a diagnosis of osteoporosis at a primary care clinic. This case underscores the challenges in predicting the level of stress required to induce Takotsubo, highlighting individual variability in stress responses. It suggests the potential utility of predictive tools for risk stratification and tailored interventions. Clinicians should recognize that stressors capable of triggering adverse outcomes are not limited to significant life events. Any stressor, regardless of how minor it may appear, can provoke a severe reaction depending on the individual’s perception and emotional response.

Common emotional triggers like the death of a loved one or surviving an accident are intuitively intense and capable of causing a catecholamine surge sufficient to induce Takotsubo. However, this case illustrates that even seemingly minor events, such as receiving a medical diagnosis, can lead to the condition. The determining factor is not the apparent severity of the stressor but the patient's subjective reaction to it. While diagnoses like hypertension, diabetes, or osteoporosis are often considered routine by clinicians, they can be life-changing and distressing for patients hearing them for the first time. Such news can provoke significant emotional stress, leading to adverse health outcomes. The medical setting itself can be a source of anxiety, as evidenced by phenomena like white coat hypertension [12]. Poor communication by healthcare providers may exacerbate this stress, particularly if information is delivered without empathy or in overly technical language, leaving patients confused, fearful, and distressed.

Our case suggests that clearer, more empathetic communication might have prevented the emotional stress that led to Takotsubo cardiomyopathy. Clinicians are encouraged to adopt frameworks such as SPIKES (Setting, Perception, Invitation, Knowledge, Empathy, Summary) not only for delivering bad news but also for communicating new chronic diagnoses. Providing patients with educational materials, referring them to support groups, and addressing their concerns can be particularly beneficial for high-risk individuals, such as postmenopausal women with pre-existing anxiety disorders. Integrating psychologists or counselors into care teams and offering close follow-up-whether virtual, telephonic, or in person-may further reduce the risk of stress-induced health events.

On average, patients hospitalized with Takotsubo cardiomyopathy spend 6.6 ± 6.2 days in the hospital [13] and often undergo invasive coronary catheterization to rule out obstructive coronary disease. Between 2010 and 2014, hospitalization costs in the United States ranged from approximately $28,465 in the South to $40,217 in the West [14]. These procedures are not without risks, making preventive interventions like improved communication and psychosocial support cost-effective alternatives.

## Conclusions

Although this case cannot be generalized, it highlights the importance of recognizing the interplay between emotional stress and physical health, especially in susceptible populations such as postmenopausal women. The presentation of Takotsubo cardiomyopathy in this 73-year-old patient was triggered by psychological stress related to her osteoporosis diagnosis and the associated fear of fractures, despite no significant life-threatening events or major predisposing factors for cardiovascular disease. It also underscores the need for clinicians to appreciate the subjective nature of stress triggers and their potential to cause significant physical health outcomes, thereby calling attention to the role of effective, empathetic communication in mitigating patient anxiety and improving outcomes. By adopting structured communication strategies (such as the SPIKES protocol), providing education, and integrating psychosocial support into patient care, clinicians can better address emotional distress and reduce the risk of stress-induced conditions like Takotsubo cardiomyopathy.

This case also emphasizes the importance of recognizing that diagnoses that clinicians may consider routine can be perceived as non-routine and profoundly stressful by patients. This highlights the need for a patient-centered approach to healthcare that prioritizes emotional well-being alongside physical health. Furthermore, future research should focus on identifying predictors of stress-induced health events, such as Takotsubo cardiomyopathy, especially in ambulatory care settings.
